# Modulation of disease-associated gut microbiota by *Schisandra chinensis*: a literature review

**DOI:** 10.3389/fphar.2026.1721950

**Published:** 2026-04-17

**Authors:** Zhimei Liu, Caiwen Wang, Hui Fan, Yifei Yue, Tianxia Sun, Liping Sun

**Affiliations:** 1 Changchun University of Chinese Medicine, Changchun, Jilin, China; 2 The Affiliated Hospital to Changchun University of Chinese Medicine, Changchun, Jilin, China; 3 The 3rd Affiliated Hospital of CCUCM, Changchun, Jilin, China

**Keywords:** Chinese herbal medicine, gut microbe, mechanism, pharmacological activate, *Schisandra chinensis*

## Abstract

**Background:**

To clarify the mechanism by which *Schisandra chinensis* regulates the disease-related gut microbiota and to identify potential directions for future research.

**Methods:**

Relevant literature published between 2020 and 2025 was retrieved from Google scholar and NCBI PubMed. Two authors screened the literature based on titles and abstracts, followed by full-text screening by another two authors to ensure the inclusion of eligible studies.

**Results:**

We found in 61 studies that *S. chinensis* rich in polysaccharides and lignans, which are primary bioactive compounds involved in modulating gut microbiota composition. These compounds have been extensively studied for their ability to rebalance gut microbiota, repair the intestinal barrier function, increase short-chain fatty acids levels, and regulate metabolic pathways. Therefore, *S. chinensis* alleviates the symptoms caused by ulcerative colitis, antibiotic-associated diarrhea, alcohol-associated liver disease, type 2 diabetes mellitus, Alzheimer’s disease, non-alcoholic fatty liver disease, systemic inflammation, and neurobehavioral alterations associated with mental illness. Additionally, another study suggested that lignans influence bile acid production by modulating gut microbiota.

**Conclusion:**

Current research on *S. chinensis* regulate gut microbiota, which have demonstrated therapeutic effects in disease. However, the precis mechanisms of *S. chinensis* regulated the gut microbiota remain unclear, and high-quality clinical trials are lacking to validate the efficacy of *S. chinensis* in humans.

## 1 Introduction

*Schisandra chinensis* (Turcz.) Baill. (Schisandraceae), the dried ripe fruit used in traditional Chinese medicine and commonly known as “Wu Wei Zi” is mainly distributed in Heilongjiang, Jilin, and Liaoning provinces, as well as Inner Mongolia Autonomous Region (Hu et al., 2012). In the history of traditional Chinese medicine, *S. chinensi*s was classified as an “upper-grade drug” in Shennong’s herbal Classic of Materia Medica. It was named “Wu wei zi” because the peel and flesh are sweet and sour, kernel is pungent and bitter, and overall tasted also includes saltiness. In traditional Chinese medicine theory, *Schisandra chinensis* is warm in property and enters the lung, heart, and kidney meridians. Traditionally, it has been used to enrich yin and promote fluid production, moisten and support the lung function, alleviate spontaneous sweating, tonify the kidney and stabilize essence, strengthen the intestines to relieve diarrhea, and nourish the heart to tranquilize the mind. It exerts effects of astringency, consolidation, tonifying qi and promoting fluid production, nourishing the kidney and tranquilizing the mind (Liu and Wang, 1999).

Luo et al. (2019) in certain regions of China, the leaves of *S. chinensis* were commonly consumed as a wild vegetable, and processed into health-promoting beverages due to their hepatoprotective activity (Bing et al., 2025). Furthermore, in Traditional Chinese Medicine clinical practice, *S. chinensis* is often prescribed for palpitations, insomnia, chronic cough due to lung deficiency, deficiency-asthma caused by the Kidney’s failure to receive qi, and collapse syndromes related to severe yin deficiency (Yuan and Li, 1997). Beyond China, *S. chinensis* has also been traditionally used in Korea, Russia, and Japan (Kim et al., 2019; Yang, 2022). In Korea, it has been applied to lower blood pressure, blood glucose, and cholesterol, as well as to treat coughs, hepatitis, bronchitis, and alcohol detoxification largely attributed to its high content of anthocyanins and other polyphenols. In Japanese Kampo medicine, it has been recorded for its antiviral, anti-inflammatory, antitussive, and hepatoprotective effects.

Although multi-compound formulations are commonly studied in Traditional Chinese Medicine, clinical evidence suggests that *S. chinensis* contributes significantly to therapeutic efficacy. Thus, its pharmacological effects are considered to involve multiple ingredients, multiple targets, and multiple mechanisms. With the aid of modern analytical techniques, numerous bioactive substances have been identified in *S. chinensis*, including lignans, polysaccharides, triterpenoids, polyphenols, organic acids, essential oils. These constituents exert therapeutic effects against a variety of diseases (Table 1), with lignans and polysaccharides being the most abundant. There are many studies focus on pharmacological effects and action mechanisms of lignans and polysaccharides. Some studies report that lignans and polysaccharides can regulate the gut microbiota.

**TABLE 1:** Pharmacological effects of the main compounds of *Schisandra chinensis*.

Compounds	Main substances	Major roles	References
Lignans	Schisandrin; Schisandrin (A-C, E); Gomisin (A-H, J, K3, N, O, R-T); Isoschizandrin; Benzoylgomisin H, Q, O; Deoxyschisandrin; Angeloylgomisin H, Q, O; Schisandroside (A-E); Neglschisandrin E; Schinlignan A-D, F, G; Schisanchinin A-D; Schischinone; Methylgomisin O; Wuweilignan E; Schineolignin A-C	Antioxidant; Anti-Inflammatory; Antiviral; Hepatoprotective; Neuroprotective; Antidiabetic; Anticancer; Cardioprotective; Anti-Platelet Aggregation; Anti-Hepatitis B Virus; Anti-Acetylcholinesterase	Co et al. (2024) ; Xiang et al. (2022) ; Yang and Yuan (2021) ; Ehambarampillai and Wan (2025)
Polysaccharides	SPJ; SCP-(1, 2, 3-1, 2-1, 22, 0-1, Ia, IIa, a, b, c, BII); SCP; SCFP-1; SCGP; WASP-(30,60,80); SCAP; SCP; SCAP-2; WSP-(N, 1, 2, 3); SP; SCPP11; SC-2; BPS(1-1, 11); SFP; ESCP; WSLSCP; ASPS-(a-1, b-2, b-3)	Anti-Viral; Anti-Tumor; Resist Fatigue; Anti-Oxidant; Immunoregulation; Anti-Inflammatory; Hepatoprotective; Relieve A Cough; Anti-Diabetic	Ji et al. (2024)
Triterpenoids	Schinchinenlactone A-C; Henrischinin C; Schinchinenin G and H; Henrischinin A-B; Schinchinenin A-F; 2β-hydroxymicrandilactone C; Wuweizidilactone J-M, O-P; Propindilactone Q, A; B; Preschisanartanin E, F, K, L-N; Arisanlactone C; Schicagenin A-C; Schisdilactone J; Isoschicagenin C; Schisdilactone A-I; Schinesdilactone A; B; Schindilactone H-K; Arisanlactone B; Wuweizidilactone I; Schindilactone H; Wuweizidilactone S; Schindilactone LM; Schinchinelactone D	Neuroprotective Effect; Immunomodulation Effects; Anti-Oxidation; Antimicrobial	Yang and Yuan (2021) ; Xu et al. (2023)
Polyphenols	Include: Flavonoids and phenolic acids: Chlorogenic; Cryptochlorogenic; Gallic; Neochromogenic; Protocatalytic; Vanillic Acids; P-Hydroxybenzoic; Protocatechuic; Salicylic; Syringic Acids; Galangin; Hesperetin; Resveratrol; Kaempferol; Quercitrin; Isoquercetin; Glucosides; Galactosides; Rutinosides; CyXylGlu; CyGluRutin; CyRutin; CyXylRutin	Antioxidant; Anti-Inflammatory; Regulating Lipid Metabolism	Skalski (2025)
Organic acids	Citric acid; Malic acid; Shikimic acid; 6-methyl citrate; Dimethyl citrate; Pentanoic acid; 4-oxo-methyl ester; Butanedioic acid; Dimethyl ester; Pentanedioic acid; Hexadecanoic acid, methyl ester; 9-octadecenoic acid, methyl ester; 8,11-octadecadienoic acid, methyl ester; 3-acetoxy-3-hydroxy; Propionic acid, methyl ester; Ethanedioic acid, dimethyl ester; Pentanoic acid, 4-oxo-methyl ester; Butanedioic acid, dimethyl ester; Pentanedioic acid, 3-oxo-dimethyl ester; 2-propenoic acid, 3-(4-hydroxy-3-methoxyphenyl)-, methyl ester; Tetracosanoic acid, methyl ester; Levulinic acid	Antibacterial; Strengthen The Human Immune System; Protect The Heart	Skalski (2025) ; Jia et al. (2023)
Essential oils	α-Yilanene; p-cymene; γ-terpinene; α-teprenone; β-laurene; sabinene, β-pinene; β-Himachalene; Gibberene D; (−)-β-elemene; (E)-β-ocimene; Lignan; α-Farnesene; Methyl acetate; 1-octene; Ethyl butanoate; citral	Anti-Inflammatory; Antioxidant; Liver Protection	Wang S. Y. et al. (2025); Zhao et al. (2022)

The human gut microbiota comprises archaea, viruses, fungi, and bacteria (Liu et al., 2022). Among bacterial phyla, *Firmicutes*, *Bacteroidetes*, *Actinobacteria*, *Proteobacteria*, and *Verrucomicrobia* are the most common, with *Bacteroidetes* and *Firmicutes* together comprising over 90% of the total gut microbial population (The Human Microbiome Project Consortium, 2012). Over long-term co-evolution, the gut microbes have formed a close symbiotic relationship with humans. In a healthy state, the gut microbiota maintains a dynamic balance of mutual control with the host organism. This homeostasis plays an important role in the physiological functions. When the gut microbiota becomes imbalanced and the intestinal barrier disrupted, this dysbiosis is considered a major contributor to autoimmune and metabolic diseases (Al Bander et al., 2020). Evidence indicates that microbial imbalance contributes to the transition from inflammatory bowel diseases (IBD) to colorectal cancer, and is also implicated in the pathogenesis of allergies, neurological diseases, obesity, enteritis, hepatic dysfunction, and immune dysregulation (Huang et al., 2021; De Filippis et al., 2021; Mann et al., 2024; Wang Z. et al., 2024; Zeng et al., 2024; Aron-Wisnewsky et al., 2021; Wang et al., 2021). Recent studies have widely recognized that diet is one of the main factors regulating the gut microbiota (Simões et al., 2022). In particular, plant-derived compounds play a vital role in preserving microbial homeostasis and promoting overall health (Liu et al., 2023). These bioactive substances in plants not only inhibit harmful microorganisms, but also influence gene expression of gut microbiota and enhance the production of beneficial metabolites (Howard et al., 2024), such as short-chain fatty acids (SCFAs). SCFAs regulate immune responses, repair the intestinal barrier to reduce systemic inflammation, and inhibit brain inflammation, thereby alleviating cognitive decline and neurological disorders. Additionally, the bile acid fermented by gut microbiota regulates the farnesoid X receptor signaling, influencing metabolism and immunity (Zhao et al., 2025; Jiang et al., 2025; Chen et al., 2023; Zhao et al., 2023, Sun et al., 2023a). More studies have demonstrated that dietary plant compounds can modulate metabolism and immunity by modulating gut microbiota (Grant et al., 2024).

*Schisandra chinensis* is a traditional herb used both as medicine and food. It is rich in bioactive compounds, including lignans, polysaccharides, triterpenoids, polyphenols, organic acids, and essential oils, each with different pharmacological activities (Table 1) (Yang and Yuan, 2021; Ehambarampillai and Wan, 2025; Ji et al., 2024; Xu et al., 2023; Skalski, 2025; Jia et al., 2023; Wang S. Y. et al., 2025; Zhao et al., 2022; Xiang et al., 2022). The pharmacological effects of *S. chinensis*, such as anti-inflammatory, antioxidant, neuroprotective, antidiabetic, anticancer, cardioprotective effects, have been well documented (Yang and Yuan, 2021; Yan et al., 2021). With growing attention to the role of gut microbiota in disease, the interaction between *S. chinensis* and gut microbiota has attracted increasing research interest. In recent years, several studies have reported that *S. chinensis* can regulate the gut microbiota related to diseases, thereby advancing understanding of gut microbiota-disease interactions.

Diet is a well-established determinant of gut microbial composition and function. Diets rich in plant-derived foods favor the production of short-chain fatty acids (SCFAs), which play critical roles in host-microbe interactions by preserving intestinal barrier integrity, maintaining gastrointestinal homeostasis, and modulating immune responses and inflammatory suppression (Vernocchi et al., 2020; Yoo et al., 2020). In contrast, disruption of the gut microbial ecosystem can contribute to the development of multiple chronic disorders, including neurodegenerative, cardiovascular, metabolic, and gastrointestinal diseases (Chen et al., 2021). Conversely, the gut microbiota exerts regulatory effects on host physiology through microbial metabolism, immune defense, and bidirectional signaling along the gut–brain axis, thereby influencing overall host health (Hajiagha et al., 2022). Within the framework of traditional Chinese medicine, herbal drugs and their bioactive extracts have been shown to restore microbial balance and reinforce the intestinal barrier, leading to improvements in inflammatory status and metabolic regulation (Che et al., 2022; Zhang et al., 2020). Notably, the intrinsic medicinal properties of herbs themselves significantly shape their modulatory effects on the gut microbiota (Zhang et al., 2021). *Schisandra chinensis*, classified as a warm-natured herbal medicine, is characterized by mild pharmacological properties and a high content of polysaccharides and lignans. These compounds can be metabolized by intestinal microorganisms, thereby promoting the generation of SCFAs and contributing to host-microbiota homeostasis.

However, to our knowledge, no systematic review on the regulation of disease-associated gut microbiota by *S. chinensis* has been published from 2020 to 2025 in Google Scholar, PubMed. To address this gap, the present review aims to synthesize current evidence and discuss mechanisms by which *S. chinensis* influences gut microbial communities and functional relevance to human disease.

## 2 Materials and methods

### 2.1 Search strategy

The literature search was conducted according to the PRISMA recommendations (Page et al., 2021). To identify studies on the pharmacological effects of *S. chinensis*, we combined plant-related terms (e.g., Magnolia vine and *S. chinensis*) with keywords referring to its bioactive constituents (such as lignans, polysaccharides, triterpenoids, polyphenols, organic acids, essential oils). To retrieve studies on the interaction between *S. chinensis* and gut microbiota, the plant-related terms above were paired with microbiome-related keywords, such as gut microbe, gut microbiome, intestinal microbiome, intestinal flora, gut flora. Literature searches were carried out across two major databases: Google Scholar and PubMed (NCBI).

### 2.2 Studies collection

The studies were selected based on the following inclusion criteria:

1. Studies published between 2020 and 2025;
2. Only original research articles presenting primary data.

**FIGURE 1:**
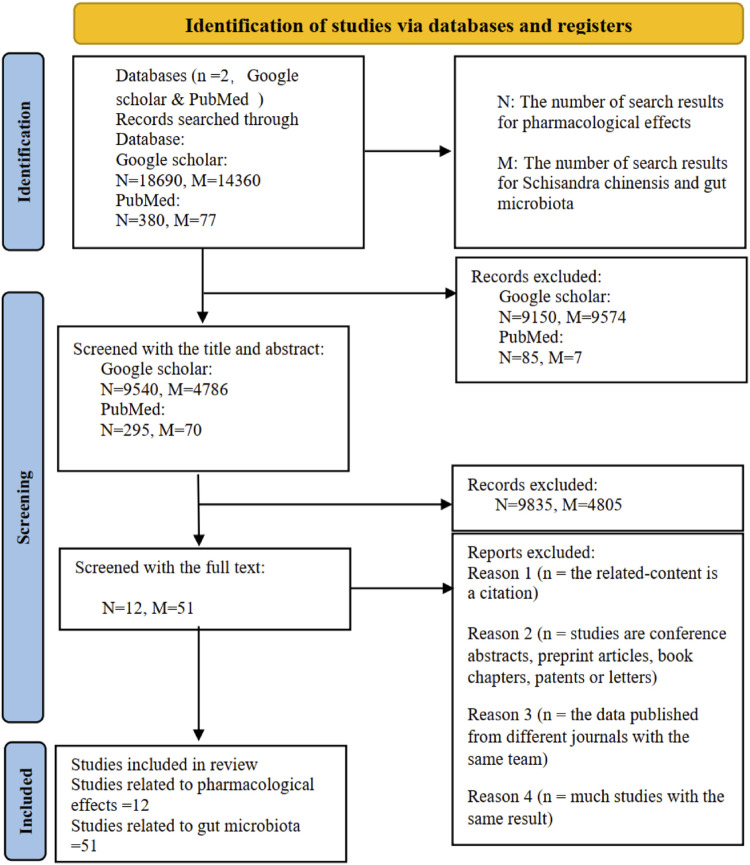
Literature search and screening process.

The exclusion criteria as follow:

1. Studies published before 2020;
2. Non-original works, such as citation or commentaries;
3. Studies are conference abstracts, preprint articles, book chapters, patents, letters;
4. The data published from the same research group with same topic in different journals;
5. Articles with *in vitro* experimental designs;
6. Studies with the same result. Figure 1 Flow diagram of study screening and selection process.

### 2.3 Studies extraction

The initial screening of studies was conducted by C.W. and H.F. based on titles and abstracts, followed by full-text assessments were performed by Y.Y. and T.S. to confirm final eligibility.

## 3 Results

### 3.1 Retrieved studies

A systematic search was performed using Google Scholar and PubMed to identify relevant studies. As shown in Figure 1, the initial search yielded 33,507 records (18,690 and 380 from Google Scholar and PubMed for pharmacological effects, respectively; 14,360 and 77 for Ziziphi Spinosae Semen and gut microbiota, respectively). After removing duplicates and irrelevant records, 14,691 records remained for title and abstract screening. Of these, 14,640 were excluded based on eligibility criteria. The remaining 51 articles underwent full-text review, and 40 were excluded because they were citations, conference abstracts, preprints, or contained duplicate data. Ultimately, 11 articles were excluded at this stage. In total, 61 articles were included in this review: 12 on pharmacological effects and 51 on the relationship between Ziziphi Spinosae Semen and gut microbiota.

### 3.2 Main active ingredients and pharmacological effects of *Schisandra chinensis*

The active ingredients of *S. chinensis*, a traditional herbal medicine, have been extensively studied. To date, approximately 86 distinct lignans, 40 polysaccharides, 83 triterpenoids, 23 polyphenolic compounds (including flavonoids and phenolic acids), 39 organic acids, and more than 85 essential oils have been identified. Most studies focused on the pharmacological activities and underlying mechanisms of lignans, polysaccharides, triterpenoids, and polyphenols, whereas organic acids and essential oils remain relatively underexplored.

The various active ingredients of *S. chinensis* demonstrate a wide range of pharmacological properties, such as antioxidant, anti-inflammatory, neuroprotective, immune regulatory, antiviral, anti-tumor, and metabolic regulatory effects (Table 1).

### 3.3 The molecular mechanism of *Schisandra chinensis* regulates the disease- associated gut microbe

#### 3.3.1 Polysaccharides

*Schisandra chinensis*-derived polysaccharides have been investigated in various disorders, including ulcerative colitis, antibiotic-associated diarrhea, alcohol-associated liver disease, type 2 diabetes mellitus (T2DM), Alzheimer’s disease (AD), and neuroinflammation. These polysaccharides are thought to act primarily by modulating gut microbial composition, thereby mitigating pathological alterations and associated clinical symptoms.

In mice models of ulcerative colitis, treatment with *S. chinensis* polysaccharide (SCP) significantly increased the relative abundance of *norank_f_Bacteroidales_S24-7_group*, *Desulfovibrio*, and *Alistipes*, while decreasing the relative abundance of *Lactobacillus*, *Turicibacter.* These microbial changes led to an elevation in level of short-chain fatty acid (SCFAs), particularly acetic acid, propionic acid, and butyric acid. This increase in SCFAs was linked to enhanced expression of *GSH*, *SOD*, *IFN-γ*, *IL-4*, along with reduced expression of *MPO*, *NO*, *ROS*, *MDA*, *TNF-α*, *IL-6*, *IL-13*, *IL-17*, and *IL-23* (Su et al., 2020; Zhang et al., 2023; Bian et al., 2022). In addition, SCP administration lowered the disease activity index, reduced tissue levels of myeloperoxidase and malondialdehyde, and decreased the abundance of *Bacteroides* and *Erysipelatoclostridium*. Conversely, it enhanced the expression of *MUC2*, *occludin*, and increased the abundance of *Muribaculaceae-unclassified*, *Lachnospiraceae- NK4A136 group* (Guo et al., 2024).

Water-soluble polysaccharides (WSPs) extracted from *S. chinensis* modulate the gut microbiota by enriching beneficial taxa have demonstrated significant modulation on gut microbial composition. WSP treatment increased the relative abundance of *Blautia*, *Intestinibacter*, *Lachnospiraceae-UCG-008*, and decreased the relative abundance of *Ruminococcus-1*, *Ruminococcaceae-UCG-014*, *Erysipelatoclostridium*. These microbial alterations promoted the production of SCFAs, particularly acetic acid and propionic acid, which in turn inhibited the NF-κB signaling pathway and reduced the secretion of pro-inflammatory cytokines such as *TNF-α* and *IL-8*, thereby alleviating intestinal inflammation (Qi et al., 2019).

In alcohol-associated liver disease, SCP increased the relative abundance of *Lactobacillus*, which facilitated the conversion of tryptophan into indoles. These metabolites activated the *aromatic hydrocarbon receptor* (*AhR*), thereby upregulating the expression of *ZO-1* and *Occludin.* Consequently, SCP treatment helped repair intestinal barrier damage, reduced LSP translocation into bloodstream, and inhibited NF-κB signaling pathway, ultimately ameliorating alcohol-associated liver disease (Chi et al., 2024).

In T2DM models, SCP restored the gut microbiota toward a normal composition by increasing the abundance of *Bacteroides* and *Alloprevotella* and reducing *Escherichia-Shigella.* These microbial changes elevated the levels of SCFAs, restored intestinal barrier integrity, and improved metabolic abnormalities and inflammatory reactions caused by T2DM (Guo et al., 2025).

In Alzheimer’s models, SCP restored the relative abundance of *Firmicutes* and *Bacteroidetes*, both typically reduced in AD, and increased the SCFAs production. These changes suppressed the activation of *NLRP*3 and NF-κB signal pathway, thereby reducing pro-inflammatory mediators and neuroinflammation, ultimately alleviating AD-related symptoms (Gao et al., 2024). SCFAs are also known to cross the blood-brain barrier, where they inhibit the microglial activation and attenuate neuroinflammation and associated metabolic disorders (Fu et al., 2023). Consistently, another study reported Schisandra polysaccharides increased the relative abundance of *Lactobacillus* and *Bifidobacterium*, which contributed to improving AD through blood-brain barrier (Sun et al., 2023b). In dementia models, SCP regulated the gut microbiota, suppressed the microglial activation, and decreased pro-inflammatory factors to alleviate dementia (Wróbel-Biedrawa and Irma, 2024). Furthermore, in broader neuroinflammatory conditions, including depression and neurodegenerative diseases, SCP restored the balance of *Firmicutes* and *Bacteroidetes*, and repaired the integrity of the intestinal mucosal barrier and blood-brain barrier, block *TLR4* receptors, inhibit the NF-κB inflammatory pathway, and reduce pro-inflammatory factors, thereby alleviate neuroinflammation and neurodegenerative diseases (Sun et al., 2023a; Du et al., 2023).

In summary, SCP exerts its regulatory effects through multiple mechanisms:

1. Modulation of gut microbiota: SCP restores gut microbiota by selectively increasing the abundance of beneficial bacteria while reducing the abundance of pathogenic bacteria. Notably, bacterial species with the same genus may be differentially regulated depending on the disease context;
2. Metabolic regulation: SCP enriches SCFAs-producing microbes, leading to elevated short-chain fatty acid levels, activation of key metabolic pathways, and enhanced intestinal barrier integrity;
3. Immune modulation: SCP suppresses the NF-κB/NLRP3 signaling pathway, promotes the production of anti-inflammatory cytokines, and mitigates systemic inflammation.

However, the regulatory effects of SCP on the relative abundance of gut microbes were different between the different diseases, and even the same disease, and this difference resulted in different downstream regulatory outcomes.

#### 3.3.2 Lignans

To date, relatively few studies have explored the role of lignans in modulating gut microbiota under disease condition. Studies primarily have focused on ulcerative colitis, non-alcoholic fatty liver disease (NAFLD), systemic inflammation, and neurobehavioral alterations associated with mental illness.

Schisandrin has been reported to re-balance the gut microbiota composition by increasing the relative abundance of *Bacteroidetes* and *Lactobacillus* while reducing *Firmicutes*, thereby enhancing the SCFAs production. These changes suppressed the TLR4/NF-κB signaling pathway and downregulated pro-inflammatory cytokines, such as TNF-α, IL-6, and IL-1β, ultimately alleviating systemic inflammation (Li et al., 2023). In contract, other studies observed that schisandrin reduced the relative abundance of *Bacteroides* while increasing *Lactobacilli*, leading to improved metabolism and restoration of intestinal barrier function (Wang et al., 2023). Schisandrin has also activated the FAK signaling pathway and regulated the gut microbiota to inhibit colitis-associated carcinogenesis. In the context of NAFLD, Schisantherin A restored gut microbiota balance by increasing the relative abundance of *Bacteroidetes* and reducing *Firmicutes*, which regulate the metabolism through metabolites of gut microbiota (Yu et al., 2022). Similarly, Yan et al. (2025) demonstrated that lignans ameliorated NAFLD through mechanisms resembling those of polysaccharides in alcohol-associated liver disease, though the microbial targets differed: lignans enriched *Muribaculaceae* and *Lachnospiraceae_NK4A136_group* while reducing *Oscillospiraceae*.

Total lignans extraction have been reported to alleviate physiological damage by increasing SCFAs levels and rebalancing gut microbiota. Observed changes included increased relative abundance of *Lactobacillus* and *Lachnospiraceae*, and decreased relative abundance of pathogenic bacteria (*Enterococcus*, *Escherichia* and *Bacteroides*), bacteria related to inflammation (*Alloprevotella* and *Bacteroides*), and *Lactobacillus* (Sun et al., 2020; Wang Z. et al., 2024; Song et al., 2021). Those alterations were associated with the suppression of the *TLR4* signal pathway and alleviation of depression-like behaviors in lipopolysaccharide-treated mice (Sun et al., 2020). Another study demonstrated that comparable microbial shifts restored intestinal barrier integrity and established a positive feedback loop with host–microbe interactions (Wang J. et al., 2024). Moreover, this alteration potentially reduced lactic acid production, which inhibited the activation of lactic acid receptors and activated the cAMP-PKA pathway to promote fat breakdown, thereby improving lipid metabolism and neuroinflammation (Yan et al., 2021). These effects ultimately alleviated depression-like behavior of mice subjected to chronic unpredictable stress. However, a study by Yan et al. (2021) reported that total lignan extraction increased relative abundance of *Lactobacillus* while reduced relative abundance of *Bacteroides*, which contrasts with the findings described above. This treatment remarkably increased SCFAs levels and regulated neuroinflammation via TLR4/NF-κB/IKKα signal pathway, leading to reduction in immobility time of mice in the open-file test and tail suspension test (Yan et al., 2021). Moreover, schisandrol B, a lignans compound, alleviated depression-like behavior by reducing the abundance of *Bacteroides fragilis* and *Lactobacillus johnsonii*, which are involved in bile acid production (Wang P. et al., 2025).

Although few studies have examined the impact of lignans on gut microbiota in type 2 diabetes mellitus (T2DM), current findings indicate that lignans may alleviate T2DM-related symptoms by increasing the abundance of *Bifidobacterium* and *Akkermansia*, while decreasing the abundance of *Ruminococcus* (Shi et al., 2025).

Overall, similar to SCP, lignans prevent the disease by rebalancing the gut microbiota, regulating metabolism, and modulating immune responses. However, the gut microbiota regulated by lignans varies across different diseases, and even among studies on the same disease, leading to diverse downstream pharmacological mechanisms.

#### 3.3.3 The pharmacological action of unidentified compounds

Several studies have reported the pharmacological effects of *S. chinensis* without clearly identifying the specific active constituents involved. *Schisandra chinensis* exerts therapeutic benefits in obesity, UC, and alcoholic hepatitis, as well as play a role in promoting spermatogenesis (Molinari et al., 2022; Cheng et al., 2024; Xiang et al., 2022; Liu et al., 2024).

Obese participants consumed 6.7 g of dried *S. chinensis* berry powder daily for 12 weeks. This intervention enriched the abundance of *Bacteroides, Prevotella, Roseburia, Bifidobacterium,* and *Akkermansia*, while reducing *Ruminococcus abundance*. *Consequently, S. chinensis* regulated microbial composition, influenced host metabolism, and improved obesity-related metabolic parameters (Molinari et al., 2022).

In another study, *S. chinensis* bee pollen extract (SCPE) exerted therapeutic effects in UC. SCPE increased the relative abundance of *Bacteroidetes*, including *Akkermansia*, *Lactobacillus*, *Roseburia*, *Prevpotella*, *Parabacteroides*, and *Mucispirillum*, resulting in a significant increase in SCFAs levels. These changes also activated G-protein-coupled receptors, promoting the production of the anti-inflammatory factor IL-10, and contributing to amelioration of inflammatory symptoms (Cheng et al., 2024).

A study investigating the total extracts of *S. chinensis* in the treatment of alcoholic hepatitis showed that the combination of polyphenols, flavonoids, triterpenoids, lignans, organic acids, and polysaccharides contributed to ameliorating alcoholic hepatitis. Interestingly, schisandrol A and schisandrin B did not exert protective effects in this model (Xiang et al., 2022). The total extract increased SCFAs and lactic acid levels, improved intestinal barrier integrity, attenuated endotoxin translocation into the bloodstream, and suppressed expression of TNF-α and IL-1β (Xiang et al., 2022).

Furthermore, unidentified effective compounds extracted from *S. chinensis* were reported to promote the conversion of tryptophan into aryl-hydrocarbon receptor (*AhR*) ligands by enriching the relative abundance of *Lactobacillus* and reducing the relative abundance of pathogenic bacteria. These gut microbiota alterations activated the *AhR* signaling pathway, regulated the expression of downstream genes (such as *Cyp1a1* and *Cyp1b1*), and increased the levels of pyruvate, lactate, and ATP in testicular tissue, thereby providing energy to support spermatogenesis (Liu et al., 2024).

In all the studies discussed above, although the specific active constituents were not identified, these findings suggest that the pharmacological effects of *S. chinensis* are mediated through regulation of gut microbiota, elevation of SCFAs levels, and regulation of metabolism and immune responses. These mechanisms are consistent with those attributed to purified lignans and polysaccharides.

## 4 Conclusion

Different compounds of *S. chinensis* extraction, such as SCP, lignans and other unidentified compounds, can alleviate diseases by regulating the gut microbiota, although their mechanisms of action differ. SCP has been shown to restore the gut microbiota balance, promote SCFAs production, repair the intestinal barrier function, and inhibit inflammation-related metabolic pathways in various disease models. Research on the role of lignans in regulating disease-associated gut microbiota remains limited; However, some studies suggest that lignans can similarly regulate gut microbiota and associated metabolic pathways, increasing SCFAs production to mitigate inflammation, metabolic dysfunction, and neurobehavioral abnormalities. However, the same compound may exert different regulatory effects on gut microbiota depending on the specific disease or even within the same disease. Furthermore, extracts of unidentified compounds, or in multi-herbal medicine containing *S. chinensis* appear the similar effects on the gut microbiota comparable to those of single-compound extracts. These observations indicate that a common therapeutic mechanism exists between single and multi-compound extracts, with key differences likely arising from the specific disease context and microbial targets.

**FIGURE 2:**
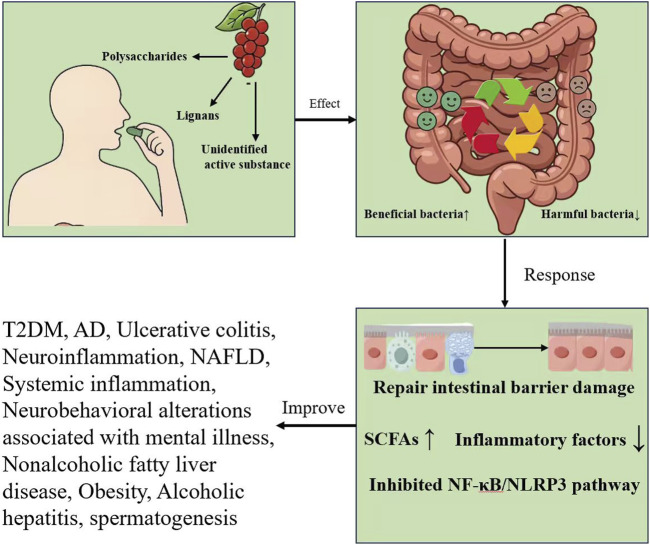
Overview of the effects of *Schisandra chinensis*.

Based on accumulated experimental evidence, a plausible microbiota-mediated mechanism has been proposed to explain the therapeutic effects of *S. chinensis*. Following dietary intake, *S. chinensis* delivers a range of bioactive constituents, particularly polysaccharides and lignans, which undergo metabolic transformation in the gastrointestinal tract. These metabolites interact with the intestinal microenvironment and are consistently associated with reshaping the gut microbial community, characterized by an increased abundance of beneficial taxa and a concomitant reduction in pathogenic populations. Restoration of microbial homeostasis is frequently accompanied by elevated levels of short-chain fatty acids (SCFAs). Importantly, across multiple disease models, increases in SCFAs are temporally followed by suppression of the NF-κB signaling pathway and the NLRP3 inflammasome, together with reduced expression of pro-inflammatory mediators. Overall, these findings suggest that the therapeutic actions of *S. chinensis* can be conceptualized within a gut–microbiota–organ axis. Collectively, these microbiota-driven effects provide a mechanistic basis for the beneficial roles of *S. chinensis* in a range of disease contexts, including ulcerative colitis, type 2 diabetes mellitus (T2DM), Alzheimer’s disease (AD), non-alcoholic fatty liver disease (NAFLD), obesity, and neuroinflammation (Figure 2).

## 5 Limitations and future research priorities

Despite these encouraging findings, several limitations remain. First, most existing studies focus on a few representative compounds, while other major bioactive constituents such as essential oils, organic acids, and triterpenoids are rarely explored in the context of gut microbiota. Second, the mechanistic understanding of how *S. chinensis*-derived metabolites interact with specific microbial taxa or host signaling pathways is still limited. Third, at present, the available evidence is mainly derived from animal studies, while data from clinical investigations remain comparatively scarce; thus, high-quality clinical trials are urgently needed to validate these findings in humans. Additionally, studies often lack standardized compound identification and quantification, making cross-study comparisons difficult.

In future research, a more systematic and comprehensive investigation into the full spectrum of *S. chinensis* constituents and their microbial targets is warranted. Advanced multi-omics technologies should be integrated to elucidate the complex interactions among active compounds, gut microbiota, and host physiology. Moreover, clarifying the contribution of individual compounds in polyherbal formulations will help optimize therapeutic strategies and improve reproducibility. Ultimately, the development of well-characterized, microbiota-targeted *S. chinensis* interventions may offer novel avenues for the prevention and treatment of microbiome-associated diseases.
